# A Cross-Sectional Comparative Characterization of Hematological Changes in Patients with COVID-19 Infection, Non-COVID Influenza-like Illnesses and Healthy Controls

**DOI:** 10.3390/v15010134

**Published:** 2022-12-31

**Authors:** Mansi Kala, Sohaib Ahmad, Meghali Dhebane, Kunal Das, Manish Raturi, Meghna Tyagi, Anuradha Kusum

**Affiliations:** 1 Department of Pathology, Himalayan Institute of Medical Sciences, Swami Rama Himalayan University, Swami Ram Nagar, Jolly Grant, Dehradun 248016, Uttarakhand, India; 2 Department of Medicine, Himalayan Institute of Medical Sciences, Swami Rama Himalayan University, Swami Ram Nagar, Jolly Grant, Dehradun 248016, Uttarakhand, India; 3 Department of Pediatrics, Division of Pediatric Oncology and Bone Marrow Transplantation, Himalayan Institute of Medical Sciences, Swami Rama Himalayan University, Swami Ram Nagar, Jolly Grant, Dehradun 248016, Uttarakhand, India; 4 Department of Immunohematology and Blood Transfusion, Himalayan Institute of Medical Sciences, Swami Rama Himalayan University, Swami Ram Nagar, Jolly Grant, Dehradun 248016, Uttarakhand, India

**Keywords:** hematological changes, influenza-like illness, coronavirus infection, COVID-19, neutrophil–lymphocyte ratio

## Abstract

Introduction: Studies have documented the role of the “neutrophil-to-lymphocyte ratio” (NLR) in influenza virus infection. In addition, morphometric parameters derived from automated analyzers on the volume, scatter and conductivity of monocytes, neutrophils and lymphocytes in many viral etiologies have helped with their early differentiation. With this background, we aimed to characterize the hematological changes of coronavirus-positive cases and also compare them with the healthy controls and patients affected by non-COVID Influenza-like illnesses so that early isolation could be considered. Material and Methods: This was a cross-sectional analytical study carried out in the years 2020–2022. All cases with COVID-19 and non-COVID-19 Influenza-like illnesses and healthy controls above 18 years were included. Cases were diagnosed according to the WHO guidelines. All samples were processed on a Unicel DxH 800 (Beckman Coulter, California, USA) automated hematology analyzer. The demographic, clinical and regular hematological parameters along with additional parameters such as volume, conductivity and scatter (VCS) of the three groups were compared. Results: The 169 COVID-19 cases were in the moderate to severe category. Compared with 140 healthy controls, the majority of the routine hematological values including the NLR (neutrophil-to-lymphocyte ratio) and PLR (platelet-to-lymphocyte ratio) showed statistically significant differences. A cutoff of an absolute neutrophil count of 4350 cell/cumm was found to have a sensitivity of 76% and specificity of 70% in differentiating moderate and severe COVID-19 cases from healthy controls. COVID-19 and the non-COVID-19 Influenza-like illnesses were similar statistically in all parameters except the PLR, mean neutrophilic and monocytic volume, scatter parameters in neutrophils, axial light loss in monocytes and NLR. Interestingly, there was a trend of higher mean volumes and scatter in neutrophils and monocytes in COVID-19 cases as compared to non-COVID-19 Influenza-like illnesses. Conclusion: We demonstrated morphological changes in neutrophils, monocytes and lymphocytes in COVID-19 infection and also non-COVID-19 Influenza-like illnesses with the help of VCS parameters. A cutoff for the absolute neutrophils count was able to differentiate COVID-19 infection requiring hospitalization from healthy controls and eosinopenia was a characteristic finding in cases with COVID-19 infection.

## 1. Introduction

Coronaviruses (CoVs) are positive-sense single-stranded RNA. Many lives are being succumbed to COVID-19 infection all over the world. Therefore, an understanding of the interaction between COVID-19 infection and the immune mechanism could help in managing patients requiring hospitalization [1].

COVID-19 viral infection initially involves the epithelium of the upper respiratory pathway, eventually leading to potential alveolar damage. The subendothelial collagen activates the platelets, at the same time monocytes and neutrophils are attracted towards the site of damage. Together these promote inflammation and thrombosis in the small vessels. Macrophages in the alveoli acquire the inflammatory phenotype, which may cause pyroptosis. It may cause programmed cell death of T cells. It is seen that CD16+ T cells and natural killer (NK) cells with a high level of cytotoxic protein may have an association with severe disease [2].

Neutrophils are the earliest cell mobilized at the site of infection and show the first response. Neutrophils are known to control bacterial infection; however, their role in eliminating viral infection is also equally important [3]. Endosomal TLR7 in neutrophils helps in eliminating influenza virus infection from the body with enhanced phagocytosis [4]. The literature has suggested the role of neutrophils in COVID-19 pathogenesis in blood as well as tissues [5,6]. Neutrophils are the first to respond in a viral infection and show antiviral properties, but the neutrophil extracellular trap (NET) formation could be harmful. Data suggest the role of neutrophils is diverse in COVID-19, which may be protective initially and detrimental later [2]. There are qualitative changes in monocytes that show a predominance of classical monocytes in patients admitted to ICU with a COVID-19 infection. In fact, classical monocytes also constitute 80–95% of circulating monocytes [7,8,9].

The monocytes are also shown to express high HLADR in mild COVID-19 infections, whereas HLADR expression was high in severe cases with COVID-19 infection, suggesting monocyte dysfunction. The role of epithelial cells and monocyte-derived IL -6 and IL-1β, respectively, have emerged in COVID-19 infection [2].

In COVID-19, T lymphocytes are sent to the site of infection followed by cytokine production from macrophages, neighboring endothelial cells and epithelial cells; helper T cells activate cytotoxic T and B lymphocytes, which help in killing viruses and the production of antibodies, respectively, suggesting that the presence of cytotoxic T cells could be protective against COVID-19 infection. NK cells are a part of our innate immunity and studies suggest COVID-19 interferes with the functioning of NK cells [10,11].

It has also been suggested that an increased number of adaptive NK cells containing cytokine proteins are associated with severe disease. The lung parenchymal tissue affected by fatal COVID-19 show a predominance of dendritic cells, macrophages and NK cells with no increase in T cells. Lack of CD 8 T cells has been correlated with fatal outcomes. Severe cases have also shown higher levels of pro-inflammatory macrophages or classically activated M1-like macrophages, as well as reduced levels of myeloid dendritic cells (mDCs) and plasmacytoid dendritic cells (pDCs) in the lung parenchyma [2].

Volume conductivity and scatter (VCS) technology in hematology analyzers has revolutionized hematological parameters. The values derived for the volume, conductivity and scatter parameters of different leucocytes has helped us to find its role in early identification of sepsis, dengue and scrub typhus [12]. It uses the direct current impedance for calculating the cell counts as well as the volume of all cell types. Radio band frequency used in evaluating the internal complexity of the cell and the laser beam used to measure light from various angles including the forward scatter provides the morphometry of these cells in the form of cell population data [13]. With this background, we hypothesized that studying these morphometric data of the circulating leucocytes with the help of an automated analyzer could be quantified and may serve as a biomarker in not only detecting COVID-19 infections but also differentiating them from other non-COVID Influenza-like respiratory illnesses.

## 2. Material and Methods

This is a cross-sectional analytical study comprising all adults (over 18 years) presenting to a single tertiary referral center in the north Indian state of Uttarakhand with fever and respiratory symptoms in the years January 2020–August 2022. The Indian Council of Medical Research (ICMR)-approved COVID-19 rapid antigen test (RAT) and COVID-19 RT-PCR were performed in all cases. All patients with clinical suspicion and with either or both COVID-19 RAT and COVID-19 RT-PCR test positivity were considered COVID-19 positive cases [14]. Non-COVID-19 influenza-like respiratory illnesses were further categorized. Thus, if patients that were deemed under suspicion for infection and tested negative for COVID-19 using the COVID-19 rapid antigen test, COVID-19 RT-PCR and bacterial cultures of respiratory specimens were deemed negative [15,16]. A group of age- and gender-matched, healthy controls were included. These controls were healthy, without a fever or respiratory symptoms, and were enrolled for a general health check-up. Patients who were inadequately worked up for molecular and bacterial cultures and with missing hematological parameters were excluded from our study. All the relevant demographic and clinical details of the patients including age, gender, duration of symptoms, charting of respiratory rate, oxygen saturation on pulse oximetry and the local and systemic examination findings were recorded. Peripheral blood EDTA samples were analyzed on a Unicel DxH 800 (Beckman Coulter, CA, USA) automated hematology analyzer. The VCS data included volume, conductivity and scatter parameters of the three groups, namely COVID-19, non-COVID-19 influenza-like respiratory illnesses and healthy controls, which underwent comparative analyses. The demographic, clinical and VCS data including volume conductivity and scatter parameters of the three groups, namely COVID-19, non-COVID-19 influenza-like respiratory illness and healthy controls, were compared. The normality of the data was determined by using a one-sample Kolmogorov–Smirnov test. Continuous variables were expressed in terms of frequency or median (P25, P75) and were compared using an unpaired Students’ t test or the non-parametric Mann–Whitney test or Kruskal–Wallis test, depending upon the normality. Categorical variables were expressed in terms of frequency and percentages and were compared using χ2 statistics or Fisher’s exact test. A *p*-value <0.05 was considered as statistically significant. Receiver operating curve (ROC) analysis was performed to determine the efficacy of various parameters, distinguishing COVID-19 from other respiratory viral infections and also from the healthy control group.

## 3. Results

169 patients with COVID-19 infection, 113 patients with non-COVID-19 influenza-like respiratory illnesses and 140 healthy controls were included in this study. The mean age (in years ± S.D.) was higher in COVID-19 (55 ± 14.6) than in non-COVID-19 influenza-like respiratory illnesses patients (50 ± 16.5) as assessed against the healthy controls (38.14 ± 8.7), and overall, males constituted the majority of the study subjects across all the three groups (72%). Total leucocyte count was inconsistent and ranged from leucopenia to leukocytosis. Out of 169 COVID-19 cases, 7 had leucopenia, 50 had leukocytosis, 50.88% (86/169) had neutrophilia. Neutrophilia was present in both groups of patients, whereas eosinopenia was seen in 52.66% (89/169) with a COVID-19 infection. Eosinopenia was seen exclusively in patients with COVID-19. Of all the symptomatic patients, only five cases tested negative for COVID-19 using the RAT and positive using an RT-PCR. For the rest, all the cases were concordantly positive with both RAT and RT-PCR.

Table 1 compares the demographic and hematological parameters of COVID-19, non-COVID-19 Influenza-like illnesses and healthy controls. The COVID-19 group significantly differed from the healthy controls in NLR, PLR, mean volumes of monocytes, neutrophils and lymphocytes, mean lymphocyte conductivity and monocyte and lymphocyte scatter. Likewise, COVID-19 and the non-COVID-19 Influenza-like illnesses were statistically similar in all parameters except in PLR, mean neutrophilic, monocyte volume, a few scatter parameters in neutrophils and axial light loss in monocytes and NLR. All values were lower in non-COVID-19 Influenza-like illnesses in comparison to COVID-19.

**Figure 1:**
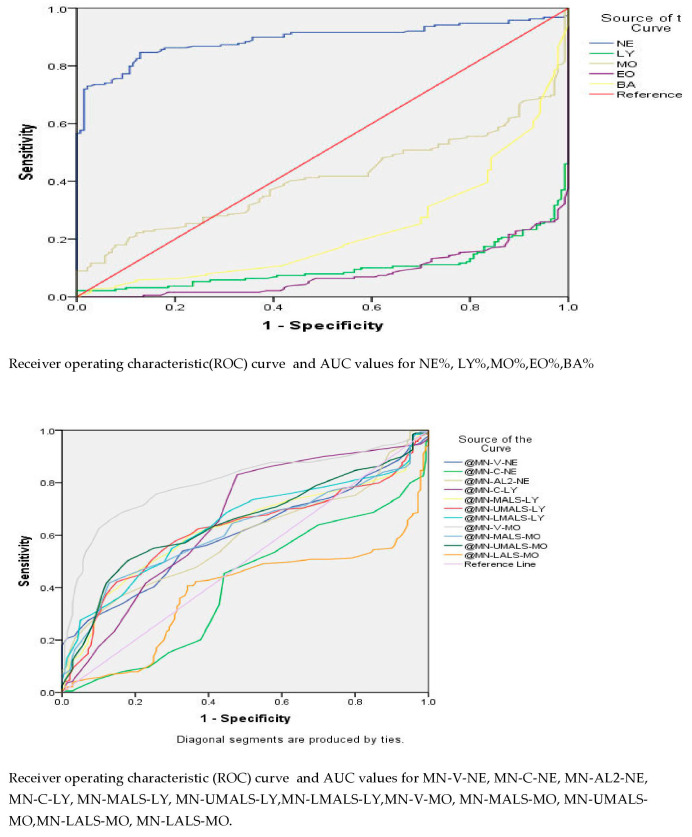
ROC curve for COVID-19 against healthy controls.

**Figure 2:**
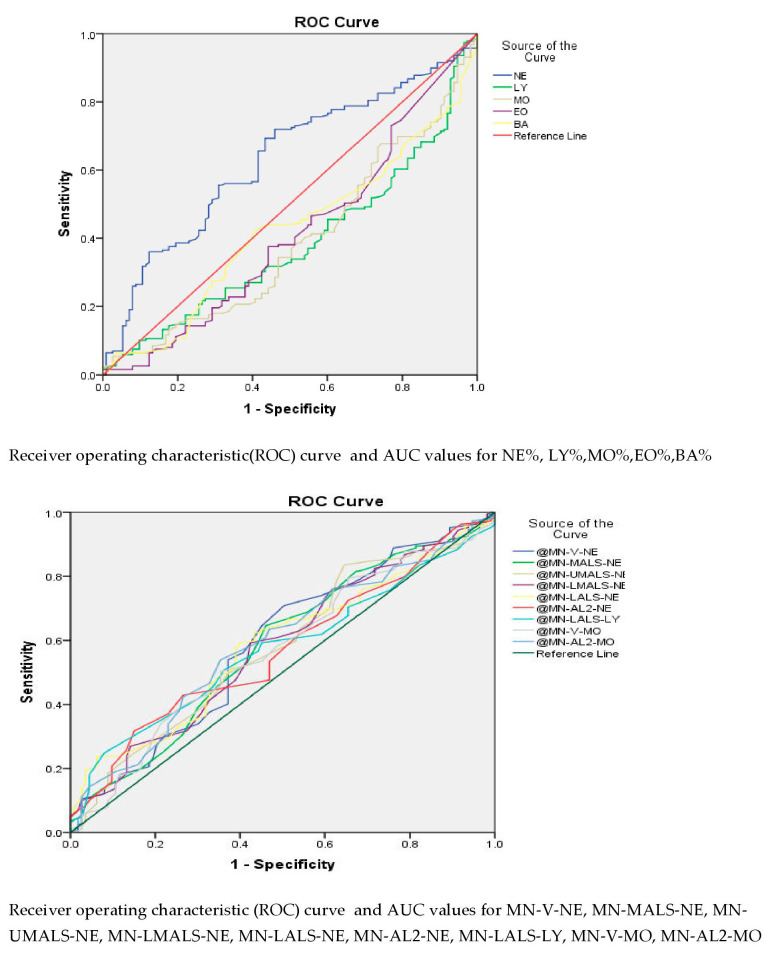
ROC curve for COVID-19 against non-COVID-19 Influenza-like illnesses.

In comparing COVID-19 and healthy controls (Table 2; Figure 1), we observed an absolute neutrophil count of 4350 cell/cumm and mean monocyte volume of 174.5, which showed an area under the curve of 0.816 with a CI of 0.769 to 0.863 and 0.798 with a CI of 0.749 to 0.846, respectively, with high statistical significance. However, no statistically significant cutoff differentiating COVID-19 from non-COVID-19 Influenza-like illnesses was seen (Table 3; Figure 2).

## 4. Discussion

We observed that the patients’ ages were higher in both illness groups, with mean ages of 55 ± 14.6 and 50 ± 16.5 years, respectively. There was a preponderance of males in all the groups. Interestingly, the 20–49 years of age group was most affected in India. These findings were also in consensus with many previous studies, which showed a male preponderance and higher population of deceased above the age of fifty years [17]. In our study, there was a wide range of total leucocyte count (TLC) ranging from leukopenia to leukocytosis in cases of COVID-19, which has been observed in previous studies as well [18]. Absolute neutrophilia was present both in patients with COVID-19 and non-COVID Influenza-like illnesses in our study. The same finding has been observed in many studies [19,20,21]. Our study found a cutoff of 4350 cell/cumm with a sensitivity and specificity of 76% and 70%, respectively, which was able to differentiate COVID-19 subjects requiring hospitalization from healthy controls. Therefore, in a suspected case of COVID-19, an absolute neutrophil count greater than 4350 cell/cumm was able to differentiate it from healthy controls, suggesting the role of neutrophils in a COVID-19 infection.

Neutrophils have primary and secondary granules. Normally, these primary granules are unapparent in neutrophils; however, with a premature release, these primary granules may retain their affinity for stain and may appear as dense granulations, which are called toxic changes [22]. Thus, the scatter of neutrophils may not correlate with toxic changes apparent on staining. Eosinopenia was seen in patients with COVID-19 but not in non-COVID Influenza-like illnesses and healthy subjects. This feature has helped differentiate COVID-19 from non-COVID Influenza-like illnesses. This was also observed by Tanni F. et al., who declared eosinopenia as an early diagnostic tool for COVID-19 cases [23].

In our study, analysis of VCS suggested morphological changes in the leucocytes in the form of higher mean volumes in neutrophils, monocytes and lymphocytes during a COVID-19 infection when compared to healthy controls. Changes in the volumetric parameters of leucocytes have been described in sepsis and other bacterial infections, pointing toward the fact that changes in volume are a manifestation of an immune response to a severe infection. [24] It is hypothesized that the premature release of neutrophils may result in the skipping of a nuclear division and as a result may cause increase mean neutrophil volume (MNV-NE) [24,25].

Previous studies have reported monocytopenia in COVID-19 infection and qualitative changes, such as the predominance of classical monocytes in the peripheral smears in cases of COVID-19 infection [8]. However, in our study we did not find monocytopenia in COVID-19 cases as compared to healthy controls. The mean monocytic volume was higher in COVID-19 cases, but the scatter parameters were non-contributory in our study. There was increased mean lymphocytic volume, conductivity and scatter of lymphocytes in cases with COVID-19 infection as compared to healthy controls. Lymphopenia < 1100 cell/cumm was observed in patients with COVID-19 infection along with an increase in mean lymphocytic volume, conductivity and scatter of lymphocytes as compared to the healthy controls. Lymphopenia and changes in the VCS parameters of lymphocytes have been reported with viral etiology by a few studies [26,27]. The increased scatter and conductivity in lymphocytes observed in viral etiologies may hypothesize a predominance of NK cells and CD8 cells on a peripheral smear. Cytotoxic T and NK cells’ roles have been described in COVID-19, where CD8 may be protective in COVID-19 and blunting of an NK response, which may be a manifestation of a severe COVID-19 infection [10,11]. The NLR and PLR ratio was able to statistically differentiate COVID-19 patients from healthy controls in our study. The role of NLR and PLR has been described in COVID-19 and other non-COVID-19 viral illnesses by previous studies [28,29].

Sensitivity and specificity were not high for most of the parameters in order to establish a cutoff, especially when differentiating COVID-19 from non-COVID-19 Influenza-like illnesses. We observed some trends to differentiate COVID-19 infection from non-COVID-19 influenza-like respiratory illnesses. We found mean neutrophilic volume and all the neutrophilic scatter parameters and mean monocytic volumes to be higher in COVID-19 infection as compared to non-COVID-19 influenza-like respiratory illnesses. NLR was capable of differentiating COVID-19 infection from non-COVID-19 Influenza-like illnesses, statistically. This may be because of the similarity in the pathophysiology in COVID-19 and non-COVID-19 influenza-like illnesses. However more cases would have shed more of a light on possible trends when assessing monocytic and neutrophilic additional parameters.

One of the limitations of our study was the high sensitivity but poor specificity of COVID-19 testing. The non-COVID-19 influenza-like respiratory illness group had variable etiologies and thus the identification of this specific group may not be appropriate. Sensitivity and specificity of the RAT was 72.1% and 98.7% among symptomatic cases, respectively [15]. One of the limitations is co-infection as well as cross reactivity rapid Ag testing with other respiratory illnesses. Although variable and low, cross reactivity with mycoplasma pneumoniae, rhinovirus and enterovirus has been reported [30,31,32]. Co-infection with other viruses can also be a confounding factor for this study.

## 5. Conclusions

Hematological parameters showed morphological changes in COVID-19-positive cases that were documented by VCS parameters with a hematology analyzer. A neutrophilia cutoff of 4350 cell/cumm showed a cutoff differentiating COVID-19 cases from healthy controls. Eosinopenia and increased NLR also showed a trend of association with COVID-19 cases. Trends were observed in neutrophil and monocyte volumes and scatter parameters between COVID-19 cases and non-COVID-19 influenza-like respiratory illnesses.

## Figures and Tables

**Table 1:** Comparison of the demographic and hematological parameters of COVID-19, non-COVID-19 Influenza-like illnesses and Healthy Controls.

Parameters	COVID-19 (N = 169)	Control (N = 140)	Non-COVID-19 Influenza-like Illnesses (N = 113)	P^a^	P^b^
Age, Y	55 ± 14.6	38.14 ± 8.7	50.19 ± 16.5	0	0.009
Male, n (%)	134(70.9)	92(65.7)	79(69.9)	0.31	0.85
WBC (×10^9^)	9.9 ± 6.1	6.8 ± 1.4	9.06 ± 4.5	0	0.197
RBC (×10^12^/L)	4.1 ± 0.8	4.5 ± 0.5	4.1 ± 0.767	0	0.67
HB (g/L)	12.01 ± 2.2	13.8 ± 1.6	12.1 ± 2.21	0	0.85
PLT (×10^9^/L)	207.2 ± 121.5	195 ± 75.7	233.5 ± 136	0.32	0.084
NE %	75.8 ± 15.2	56.7 ± 8.05	71.5 ± 11.3	0	0.014
LY %	13.1(7.1–21.2)	30.9(26.7–36.7)	17.7(12.1–23.7)	0	0.001
MO %	6.7(4.5–8.8)	7.3(6.4–8.8)	7.9(5.3–10.2)	0.005	0.001
EO %	0.19(0.0–0.8)	2.6(1.6–4.2)	0.37(0.09–1.5)	0	0.01
BA %	0.3(0.2–0.5)	0.6(0.4–0.8)	0.35(0.29–0.52)	0	0.02
NE # (×10^9^/L)	6.4(4.7–10.5)	3.7(3.1–4.5)	6.09(3.67–8.1)	0	0.06
LY # (×10^9^/L)	1.08(0.7–1.57)	2.0(1.7–2.4)	1.2(0.93–1.8)	0	0.011
MO # (×10^9^/L)	0.5(0.4–0.74)	0.5(0.4–0.6)	0.6(0.4–0.86)	0.109	0.012
EO # (×10^9^/L)	0.007(0.0–0.1)	0.2(0.1–0.3)	0.15(0.0–0.13)	0	0.123
BA # (×10^9^/L)	0.015(0.0–0.04)	0.0(0.0–0.1)	0.02(0.0–0.04)	0.003	0.305
MN-V-NE	152(146–158)	149(146–153.7)	148(143.5–156)	0.002	0.009
MN-C-NE	145(141–147)	145(143–149)	145(143–146)	0.004	0.667
MN-MALS-NE	141(136–145)	141(137–144)	138(131–144)	0.965	0.017
MN-UMALS-NE	142(136–145)	141(139–144)	141(134–145)	0.449	0.024
MN-LMALS-NE	136(129–141)	136(131–140)	133(124–140)	0.688	0.023
MN-LALS-NE	196(146–208)	157(144–204.7)	176(141–205)	0.062	0.015
MN-AL2-NE	138(133.5–143)	136(134–140)	137(133–140)	0.006	0.042
MN-V-LY	90(86–94)	89(87–90.7)	89(85–92)	0.051	0.434
MN-C-LY	118(114–120)	113(111–118)	118(115–121)	0	0.73
MN-MALS-LY	75(67.5–79)	70(67–75)	76(72.5–78)	0	0.344
MN-UMALS-LY	80(69–86)	73(69–79)	80(77–85)	0	0.176
MN-LMALS-LY	65(60–70)	61(58–66)	66(61–68)	0	0.942
MN-LALS-LY	40(34–43.5)	36(35–43)	37(33.5–42)	0.577	0.036
MN-AL2-LY	65(61–69)	65(63–70)	64(62–68)	0.126	0.13
MN-V-MO	182(175–188)	170(167–175)	179(172–185)	0	0.047
MN-C-MO	124(121–127)	123(121–127)	124(122–126)	0.284	0.517
MN-MALS-MO	94(88–97)	90(87–94)	93(87–960	0	0.411
MN-UMALS-MO	105(98–109)	100(96–103)	104(98.5–107)	0	0.579
MN-LMALS-MO	79(72–84)	78(74–80.7)	78(72–82)	0.05	0.209
MN-LALS-MO	92(73–115)	95(90–123.5)	85(72.5–102.5)	0	0.77
MN-AL2-MO	122(115–127)	119(115.2–125.7)	118(113–125)	0.122	0.009
NLR	6.00(3.27–12.30)	1.87(1.39–2.26)	4.02(2.82–6.48)	0	0.001
PLR	14.17(7.84–30.71)	6.19(4.07–7.99)	12.55(7.17–26.47)	0	0.173

P^a^ refers to the *p* value on comparison between COVID-19 against healthy controls. P^b^ refers to the *p* value on comparison between COVID-19 against non-COVID-19 Influenza-like illnesses. WBC—white blood cells; RBC—red blood cells; HB—hemoglobin; PLT—platelets; NE %—neutrophil percentage; LY %—Lymphocyte percentage; MO %—monocyte percentage; EO %—eosinophil percentage; BA %—basophil percentage; NE #—absolute neutrophil count; LY #—absolute lymphocyte count; MO #—absolute monocyte count; EO #—absolute eosinophil count; BA #—absolute basophil count; MN-V-NE—mean neutrophilic volume; MN-C-NE—mean neutrophilic conductivity; MN-MALS-NE—mean neutrophilic median angle light scatter; MN-UMALS-NE—mean neutrophilic upper median angle light scatter; MN-LMALS-NE—mean neutrophilic lower median angle light scatter; MN-LALS-NE—mean neutrophilic lower angle light scatter; MN-AL2-NE—mean neutrophilic axial light loss; MN-V-LY—mean lymphocyte volume; MN-C-LY—mean lymphocyte conductivity; MN-MALS-LY—mean lymphocyte median angle light scatter; MN-UMALS-LY—mean upper median angle light scatter; MN-LMALS-LY—mean lymphocyte lower median angle light scatter; MN-LALS-LY—mean lymphocyte lower angle light scatter; MN-AL2-LY—mean lymphocyte axial light loss; MN-V-MO—mean monocyte volume; MN-C-MO—mean monocyte conductivity; MN-MALS-MO—mean monocyte median angle light scatter; MN-UMALS-MO—mean monocyte upper median angle light scatter,; MN-LMALS-MO—mean monocyte lower median angle light scatter; MN-LALS-MO—mean monocyte lower angle light scatter; MN-AL2-MO—mean monocyte axial light loss; NLR—neutrophil lymphocyte ratio; PLR—platelet lymphocyte ratio.

**Table 2:** Comparison of hematological parameters of COVID-19 patients and Healthy Controls.

Test Result Variable(s)	Area Under Curve	Sensitivity	Specificity	Cutoff	CI		*p* Value
					Lower Bound	Upper Bound	
NE #	0.816	76%	70%	4.35	0.769	0.863	0
MO #	0.553	62%	42%	0.44	0.491	0.615	0.109
BA #	0.587	48%	67%	0.016	0.522	0.652	0.003
NE %	0.890	86%	82%	63.34	0.853	0.928	0
MN-V-NE	0.600	59%	55%	149.5	0.539	0.660	0.002
MN-AL2-NE	0.589	53%	57%	137.5	0.529	0.651	0.006
MN-C-LY	0.659	59%	61%	46.5	0598	0.720	0
MN-MALS-LY	0.625	62%	60%	71.5	0.565	0.686	0
MN-UMALS-LY	0.618	62%	63%	75.5	0.557	0.679	0
MN-LMALS-LY	0.641	59%	64%	63.5	0.582	0.701	0
MN-V-MO	0.798	75%	74%	174.5	0.749	0.846	0
MN-MALS-MO	0.614	64%	53%	90.5	0.554	0.675	0
MN-UMALS-MO	0.648	63%	58%	101.5	0.589	0.708	0

NE #—absolute neutrophil count; MO #—absolute monocyte count; BA #—absolute basophil count; MN-V-NE—mean neutrophilic volume; MN-AL2-NE—mean neutrophilic axial light loss; MN-C-LY—mean lymphocyte conductivity; MN-MALS-LY—mean lymphocyte median angle light scatter; MN-UMALS-LY—mean upper median angle light scatter; MN-LMALS-LY—mean lymphocyte lower median angle light scatter; MN-V-MO—mean monocyte volume; MN-MALS-MO—mean monocyte median angle light scatter; MN-UMALS-MO—mean monocyte upper median angle light scatter.

**Table 3:** Comparison of hematological parameters of COVID-19 patients and non-COVID-19 Influenza-like illnesses.

Test Result Variable(s)	Area Under Curve	Sensitivity	Specificity	Cutoff	CI		*p* Value
					Lower Bound	Upper Bound	
NE %	0.630	72%	54%	72.1	0.566	0694	0.014
NE #	0.565	67%	44%	5.14	0.497	0.631	0.060
MN-V-NE	0.589	64%	54%	148.5	0.657	0.522	0.009
MN-MALS-NE	0.582	64%	54%	138.5	0.515	0.649	0.017
MN-UMALS-NE	0.578	52%	57%	141.5	0.511	0644	0.024
MN-LMALS-NE	0.578	59%	57%	134.5	0.512	0.645	0.023
MN-LALS-NE	0.584	62%	56%	184.5	0.519	0.649	0.015
MN-AL2-NE	0.570	53%	53%	137.5	0.505	0.635	0.042
MN-LALS-LY	0.572	59%	54%	37.5	0.507	0.636	0.036
MN-V-MO	0.568	53%	54%	180.5	0.502	0.635	0.047
MN-AL2-MO	0.590	60%	54%	119.5	0.524	0.655	0.009

NE %—neutrophil percentage; NE #—absolute neutrophil count; MN-V-NE—mean neutrophilic volume; MN-C-NE—mean neutrophilic conductivity; MN-MALS-NE—mean neutrophilic median angle light scatter; MN-UMALS-NE—mean neutrophilic upper median angle light scatter; MN-LMALS-NE—mean neutrophilic lower median angle light scatter; MN-LALS-NE—mean neutrophilic lower angle light scatter; MN-AL2-NE—mean neutrophilic axial light loss; MN-LALS-LY—mean lymphocyte lower angle light scatter; MN-V-MO—mean monocyte volume; MN-AL2-MO—mean monocyte axial light loss.

## Data Availability

Due to the participant consent obtained as a part of recruitment process. The data of each individual cannot be made publicly available. The individual data points are available on request of the corresponding author and laboratory information system of HIHT SRHU.
